# The climate gluing protests: analyzing their development and framing in media since 1986 using sentiment analyses and frame detection models

**DOI:** 10.3389/fdata.2025.1569623

**Published:** 2025-05-19

**Authors:** Markus Hadler, Alexander Ertl, Beate Klösch, Markus Reiter-Haas, Elisabeth Lex

**Affiliations:** ^1^Department of Sociology, University of Graz, Graz, Austria; ^2^Institute of Mechanical Engineering, École Polytechnique Fédérale de Lausanne, Lausanne, Switzerland; ^3^Department of Statistical Science, Duke University, Durham, NC, United States; ^4^Institute of Human-Centred Computing, Graz University of Technology, Graz, Austria

**Keywords:** climate protest, gluing protest, media frames, text analysis over time, natural language processing

## Abstract

Recent climate-related protests by social movements such as *Extinction Rebellion, Just Stop Oil*, and others have included actions like defacing artwork and gluing oneself to objects and streets. Using sentiment analysis and frame detection models, we analyze a corpus of all available English-language news articles in LexisNexis, with the first recorded instance of a gluing protest appearing in 1986. Our study traces the development of this protest tactic over time and addresses three central questions from social movement literature: the use of glue in protests, the geographical spread of this tactic, and the framing of these actions. We find that gluing protests were initially associated with a range of issues—including abortion, criminal justice, and environmental concerns—but in recent years have become more strongly linked to climate activism. Media coverage of these protests is predominantly negative, although public media tends to be comparatively less so. Moreover, protesters' prognostic frames—suggestions for what should be done—are relatively rare, with discourse more often centering on policy and security concerns. From a data science perspective, we explore the use of various Natural Language Processing (NLP) methods. The discussion and conclusion section highlights challenges encountered when working with our corpus and NLP models, and suggests ways to address them in future research. We also consider how recent advancements in large language models (LLMs) could refine or extend these analyses while acknowledging important concerns related to their use.

## Introduction

Over the past few years, there has been a surge in reports of individuals engaging in unusual protests—gluing themselves to artwork, streets, and other objects to protest against climate-damaging activities. A primary goal of these protests is to attract media attention, with one of the most visible examples being the 2022 occurrence when activists from the British group *Just Stop Oil* glued themselves to museum walls and defaced artworks to protest fossil fuel consumption. The media framed these actions as a new type of protest, as can be seen in the commentary “Gluing Hands To Art Masterpieces: The Latest Climate-Protest Stunt Spreading In Europe” (Rodriguez, 2022).

However, despite their prominence in public discourse, there is limited systematic research on their historical trajectory, geographical spread, and media representation. Existing studies have primarily examined these protests in the context of contemporary climate activism. Collins and Chevrette (2024) analyze how *Just Stop Oil* uses visually provocative actions, such as throwing soup and gluing themselves to artworks, to attract media attention and criticize the British government. Garland (2023) highlights that such actions generate conflicting interpretations by simultaneously raising awareness about climate issues and provoking debates about their appropriateness. Similarly, Davatkhah (2024) positions these protests within a heritage context, examining how activists strategically use cultural institutions as platforms to deliver their environmental message.

While these and other studies contribute to understanding the tactical and symbolic dimensions of recent gluing protests, they overlook a fundamental historical question: Why did it take decades for this tactic to emerge as a recognized form of protest despite the availability of adhesive technology since the 1940s? Superglue was first synthesized by Harry Coover in 1942, meaning that activists have had access to adhesive-based protest methods for over 80 years. From a social movement perspective, this raises questions about when this specific tactic became what (Tilly and Wood, 2019) describe as a “repertoire of contention,” where it has emerged and spread across countries (McAdam et al., 2003), and how successful the protesters were in promoting a specific framing of their actions in the media (Benford and Snow, 2000).

To address these questions, we apply Natural Language Processing (NLP) techniques to a corpus spanning all available English-language news articles in LexisNexis, with the first recorded instance of a gluing protest appearing in 1986. Since this type of protest strategically aims to attract media attention, analyzing media coverage provides an effective way of analyzing. By tracing both the frequency and framing of gluing protests, we examine how these actions have evolved over time and how their prevalence has shifted across different issue areas. Sentiment analysis using the SiEBERT model (Hartmann et al., 2023) is applied to assess whether media coverage portrays these protests favorably or negatively, allowing for a systematic evaluation of variation across news sources. To extract keywords pertaining to the frames, we use a supervised BERTopic model (Grootendorst, 2022). Additionally, frame detection using the mCPT model (Reiter-Haas et al., 2023) enables the classification of dominant narratives surrounding these protests, identifying how different outlets position them within broader political and social debates.

Methodologically, this paper explores the applicability of NLP models to the study of protest dynamics, highlighting both the potential and the limitations of automated text analysis for understanding the framing and reception of social movements. The subsequent section offers a brief overview of the relevant social movement literature and introduces our substantive research questions. The materials and methods section describes our corpus derived from Lexis Nexis and our specific analytical methods. The results section starts with an analysis of the developments over time, followed by a sentiment analysis, and concludes with an analysis of the frames presented in the different outlets. The discussion and conclusion section points to a few issues we encountered using our corpus and NLP models and how to mitigate them in future research. In this section, we also discuss how recent advancements in large language models (LLMs) could refine or extend these analyses while remaining attentive to their methodological constraints.

## Background literature on social movements

The gluing protests of climate activists received significant media attention in 2022 when activists began defacing famous paintings. Recent research on this activism addresses several dimensions: Collins and Chevrette (2024) considered the tactics employed by *Just Stop Oil* (JSO) to attract media attention while avoiding actual damage; Garland (2023) highlights the tension between climate messaging and debates around vandalism; Kinyon et al. (2023) analyze 38 museum protests in 2022, predominantly around COP27, led by groups affiliated with the *A22* network; and Davatkhah (2024) explores how activists use heritage spaces to amplify their message. Together, these studies reveal the strategic, symbolic, and media-driven nature of these protests, questioning their impact and reception.

Yet, as de Moor et al. (2021) point out, climate protest itself is not new, but since COP21—the Paris meeting in 2015—movements have started to focus more on direct action. A new wave of protests began in 2018 with the founding of Extinction Rebellion (XR), who focus more on disobedient action forms compared to prior mobilizations. Pickard (2019) describes such protests as follows: “Do-It-Ourselves politics is when citizens take initiatives and act politically without relying on traditional collective structures.” Describing XR protests such as “swarming (forming a temporary blockade across a road or bridge), staging die-ins with fake blood, and activists supergluing themselves to an object or building,” Pickard defines them as “non-violent direct action (NVDA) that is deliberately disruptive (to the public, the police and Parliament) with the aim of bringing attention to the issues at stake.”

Another question of interest is the spatial diffusion of these protests. While environmental concerns and climate issues are global concerns, both at the institutional and individual level (Hironaka, 2014; Hadler et al., 2024), specific tactics are usually invented in specific places and then spread to other areas. Social movement literature refers to this process as “contagion” or “diffusion” (e.g., McAdam et al., 2003) and points to various ways action can spread, such as through social networks, media, and transnational movement organizations. We consider the location of the reported protests in this regard. Related research on protest activities shows that the public is particularly active in liberal states such as New Zealand and the UK, as well as in central European countries such as Germany and Austria (Hadler and Haller, 2013). Given that public environmental actions are more common in liberal countries such as the UK, we can expect that these locations play an important role in generating this type of protest.

A distinct strand of social movement research focuses on the framing of those actions. Benford and Snow (2000) distinguish between diagnostic frames (identifying the underlying problem and its causes, and assigning blame to those responsible, e.g., “We are the last generation before the tipping points,” *Last Generation*, Germany), prognostic frames (discussing consequences or proposing solutions and accompanying action strategies to address the identified problem, e.g., Just Stop Oil, UK, *Declare Emergency*, USA; “Lower the speed limit at freeways,” *Last Generation*, Austria,) and motivational frames (mobilizing and encouraging people to take action, e.g., “Become part of the team,” *Fridays for Future*, Austria).

Among environmental movements, particularly the more “radical” ones such as *XR* or *Just Stop Oil*, mobilization typically occurs from below, using all types of frames in a bottom-up effort for social change, and activists are often outsiders to established bodies and formal institutionalized processes (Caniglia et al., 2015). Einhorn and Corrigall-Brown (2023) emphasize that the *Just Stop Oil* activists initially targeted paintings that depicted nature scenes with the goal of showing what would be lost, but shifted to famous artwork, as they generated more views on social media and more reports on traditional media. As for the prognostic frame—what needs to be done (de Moor et al., 2021) state that the focus shifted from international organizations to national governments. Furthermore, while de Moor et al. (2021) emphasize that FFF and XR had rather vague prognostic frames such as “Listen to the Science,” the current protests show simple messages such as stop the oil use, lower speed limits, or declare emergency as pointed out before.

As for mobilization, both (XR and FFF) are able to mobilize young people, even though those who take part have comparable socio-demographics to previous activists. They also use social media to mobilize and bring messages across (de Moor et al., 2021). Important in this regard seems to be that social media channels can be directly filled with content by the social movement. In contrast, traditional media can select and modify the content they cover (Entman, 1993). Here, related research has shown that the reports in right-leaning and more conservative media reports are less favorable on climate actions (Painter and Gavin, 2016; Cushion, 2022). Regarding the media framing of this direct action, we expect that the media will select certain frames, and the conservative media will report less favorably about the protests.

Our study explores the use of NLP methods to investigate the three research questions: (a) the historical emergence of gluing protests, (b) their geographical diffusion, and (c) the media framing of gluing protests over time. While previous research has addressed how the media covers climate activism (Scheuch et al., 2024; Chen et al., 2023), we could not find any studies that focus on the specific tactic of gluing, especially using NLP methods. Other studies examine event and person-oriented framing (Minnema, 2024; Hamborg, 2023), but not in the context of disruptive climate protests. Our study thus contributes new insights to the existing research on these protests, while also reflecting on the use of NLP methods for this type of research.

## Materials and methods

Our analysis is based on media reports available in the “Lexis Uni” tool of the database Lexis Nexis, which is an archive that contains news reports and other sources from many local and international media outlets. We limited our search to “News” entries. Given our focus on protests, in which individuals glue themselves to objects, we specified the search terms in the following way:

*protest* AND (“*gluing himself” or “*glueing himself” or “*glued himself” or “*glue himself” or “*glued herself” or “*gluing herself” or “*glueing herself” or “*glue herself” or “*glued themselves” or “*glue themselves” or “*gluing themselves” or “*glueing themselves”)

This specific search in Nexis results in a corpus with a total number of 13,479 reports in the period from May 1st, 1986 (when the first entry occurred) to May 23rd, 2023 (when our research project finished). The number of available sources varies over time, as new sources are entered frequently. We thus standardized the number of hits over time to consider the changing number of underlying sources. Whenever we report changes over time, we standardize the findings using the search term “*protest*”. Standardized numbers reflect the number of reports that include the upper search term relative to the number of articles that include “protest” during a given period. We, however, need to emphasize that the database as well as our search terms are biased toward English-speaking countries and reports.

The subsequent analysis of this corpus employs natural language processing methods. For sentiment analysis, we use the *SiEBERT* model (Hartmann et al., 2023). SieBERT is pre-trained to provide both accurate and reproducible off-the-shelf binary sentiment labels for any English text data. The model predicts whether a given piece of text is positive or negative, for which we assign the numerical values of +1 and -1, respectively. We then average the predictions within a given time interval. Hence, we can discover the general tendency of collections of reports and their trends over time. For keyword extraction, we use a supervised BERTopic model (Grootendorst, 2022) i.e., cTF-IDF, an n-gram range of 1–2, removal of URLs and stopwords defined in ntlk.corpus.stopwords as well as: said, us, new, one, two, also, could, would, may, say, nt, topheadline, people, and get. cTF-IDF reduces the weight of words occurring in all categories, while increasing the weight of words that occur frequently within a category. BERTopic has the benefit of being easily interpretable. For frame analysis, we use the frame detection model *mCPT* (Reiter-Haas et al., 2023). The mCPT model is pre-trained on fourteen labels according to the definition by Card et al. (2015). It has been shown to generalize well in a zero-shot setting (i.e., without fitting to the target data) in a shared task (Piskorski et al., 2023). Hence, we reason that it might also apply well to our setting. We measure the Pearson correlation coefficients with group membership (i.e., percentage of climate relatedness). Finally, we track the changes in coefficients over distinct periods or sources to find patterns. Our procedure allows us to determine whether certain frames are strongly associated with climate protests.

## Results

### The overall trend of gluing protests and the emerging environmental focus

We use the number of media reports as an indicator of the development of these protests over time. As discussed in the methods section, we report the absolute number and a standardized indicator that addresses the problem of changing numbers of sources over time. This relative measure shows whether reports on the use of gluing tactics have increased relative to reports on any protest. Yet, the media has a gatekeeping function and might deliberately select some topics and dismiss others. Hence, we cannot claim that this overview covers all the protests that have happened.

**Figure 1:**
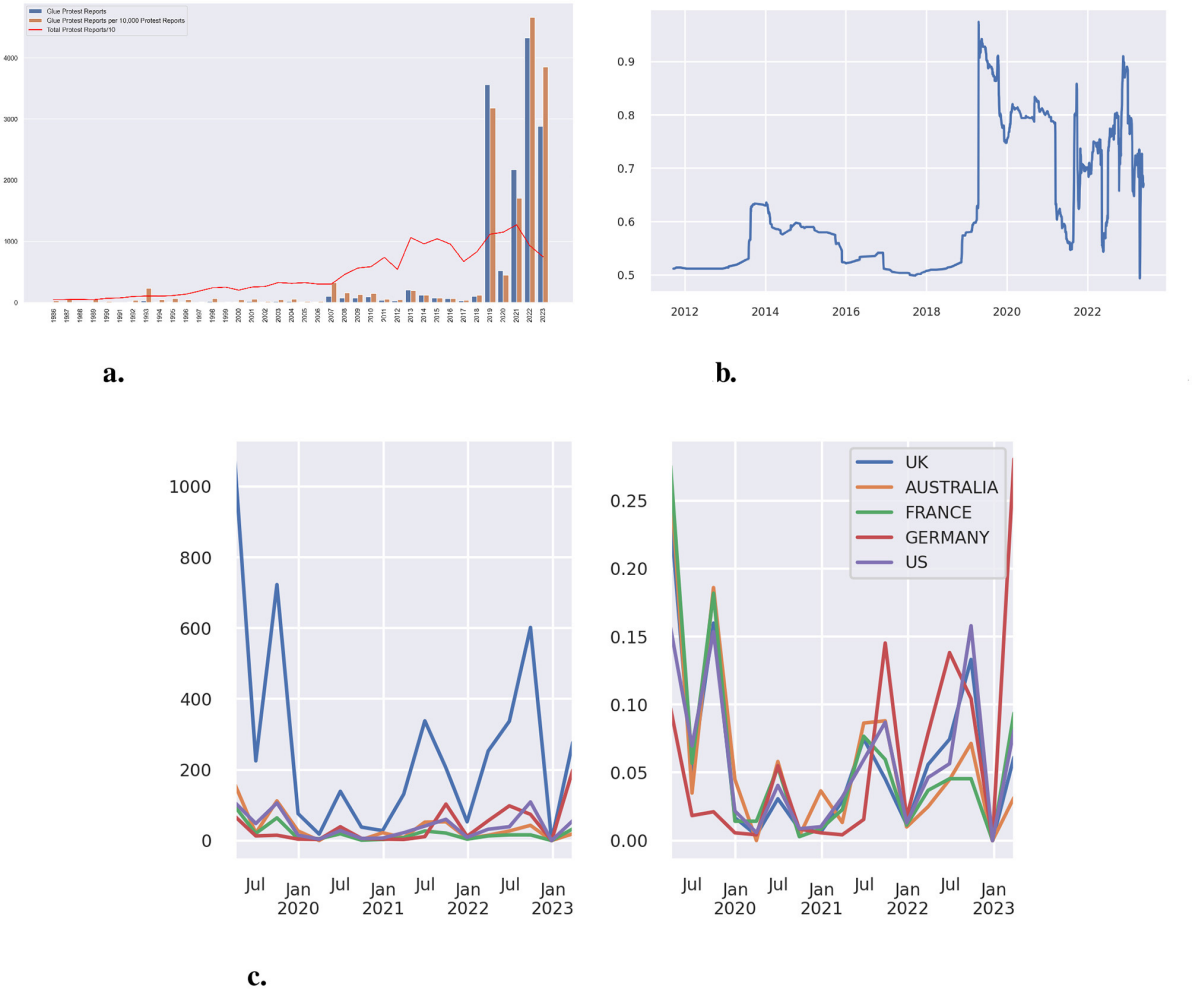
**(a)** Media reports on protests and gluing protests from 1986 to 2023. “Glue Protest Reports” shows the total for the entire year. “Total protest reports” is based on the total number of reports that include the keyword “protest” but are limited to the dates Jan 15th, Apr 15th, Jul 15th, and Oct 15th in each year. We had to restrict it to a few days, as the number of articles exceeds the search query and shows only a truncated 10,000 for any longer period. For better visualization, we have divided the total number by 10. **(b)** The prevalence of environment and climate-related topics in gluing protests (1986–2023). Percentage of articles with the keywords “climate,” “environment,” or “global warming” in our corpus. Smoothed with a period of 500. The x-value “2012” covers the entire period from 1986 to 2012, as the cumulative number of articles reached 500 only by 2012. Mind that the minimum value on the y-axis is 50%. **(c)** The location of the reported gluing protests with an environmental focus - total number (left), relative number within each country (right).

Figure 1a shows the absolute number of reports on any protest, the number of reports on protests that include our specific form of gluing protests, and the number of such reports on gluing protests relative to the absolute number of reports on any protest. In the entire Lexis Nexis database, the first entry can be found for May 1st, 1986:

“Protesters tried to get into a secretive U.S.-Australian satellite tracking base Wednesday, and 15 were arrested, police reported. (…) A police spokesman said an officer was injured when a protester who was trying to glue himself to a fence sprayed glue in his face during a struggle. Protest leaders were part of a group of 50 people cycling around Australia in a campaign against nuclear weapons, said the spokesman, who spoke on condition of anonymity. (…) ” (The Associated Press, 1986)

Similar activities happened in consecutive years in Australia. In 1987, at the Pine Gap American military base in the Northern Territory: “More than 100 demonstrators including Western Australia's Independent Senator Jo Vallentine were arrested at the Pine Gap American military base in the Northern Territory yesterday. (…) Two demonstrators superglued themselves to a surveillance tower just inside the fence. The police used solvent to free them. (…)” (Sydney Morning Herald, October 19, 1987). In 1990, it was reported that Fraser protesters who protested against logging glued themselves to machinery (Courier Mail, Aug 10, 1990). Outside Australia, we see a first entry for the United Kingdom in 1989 when it was reported that a prisoner and his girlfriend glued their hands together to protest his conviction. Similarly, in 1993, a prisoner glued himself to the gates of Buckingham Palace. In the United States, the first entries can be found in 1993 when an abortion protester glued himself to a police car, and in a report from Minnesota on the social movement Operation Rescue, which used this tactic in its protests against abortion clinics.

These protests in the early 1990s represent an initial, albeit modest, peak, which is even more apparent when considering their relative occurrence in Figure 1a. A second peak appears in 2007, coinciding with protests at the Faslane naval base and against the Trident missile program in Scotland, as well as climate-related demonstrations at Heathrow Airport, British Petroleum buildings, and other locations. From 2007 onward, reports occur regularly but remain at a relatively low level up until 2019, aside from a minor uptick in 2013 related to fracking protests. In 2019, the number of reports surged following the founding of Fridays for Future (FFF) and, in particular, the emergence of the more direct-action-oriented Extinction Rebellion (XR) movement. While climate change dominated the protest landscape in 2019—highlighted by Greta Thunberg becoming the figurehead of the FFF movement and being named Time magazine's “Person of the Year”—the focus shifted in 2020 to the COVID-19 pandemic (de Moor et al., 2021; Klösch et al., 2021). Nevertheless, overall protest reporting remained high in 2020, although references to gluing protests declined, suggesting that this tactic was rarely used in COVID-19-related demonstrations. Beginning in 2021, however, reports of direct actions began to increase again, driven by recent climate protests targeting art galleries, public streets, and other high-visibility venues.

Considering the protest's targets and goals in the different periods showed that gluing oneself to objects and other people was used for different causes. Social movements used it initially in Australia to target nuclear power plants and logging endeavors, and in the United States to target abortion clinics. In the United Kingdom, protests also occurred for rather personal reasons—against criminal charges. The question remains if, and when, this tactic was used explicitly for environmental protest. To answer this question, we calculated the proportion of articles on gluing protests that contain the keywords “climate,” “environment,” or “global warming.” Figure 1b shows that the number of articles that report glu* and protest* in the context of environmental issues is always above 50% in the depicted period. Similar to the total number of protests, we also see a small uptick in 2013 around fracking protests and a major spike in 2019, when more than 90% of the reports on gluing protests had an environmental focus.

Based on the results shown in Figures 1a, b, the tactic of gluing oneself to objects or other people was first mentioned in 1986. In the following years, a gradual increase in reports can be observed, leading to a significant surge beginning in 2019. With regard to environmental causes, instances of this tactic appear throughout the entire period, but a notable increase also occurs beginning in 2019. In sum, the period from 2019 onward can be considered a turning point — both in terms of the overall volume of reports and the growing environmental focus. The following section takes a closer look at this wave in more detail.

### The 2019 onward protest wave

Figure 1c displays the locations mentioned in media reports during the most recent period. The majority of reports refer to the United Kingdom, while other frequently mentioned countries include Australia, France, Germany, and the United States. Within-country trends show a decline in reporting on environmental gluing protests during the COVID-19 period, with more frequent reporting both before and afterward. In most countries, we observe peaks in activity preceding and following this quieter phase. Germany stands out as an exception, with protest activity intensifying in 2022 and being nearly absent prior to the COVID-19 period.

**Figure 2:**
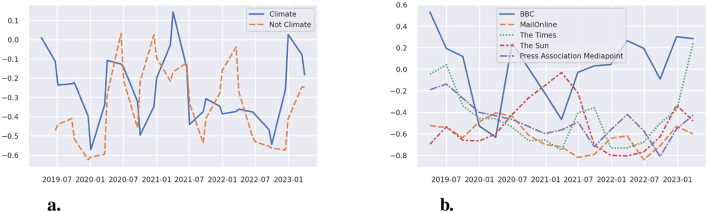
**(a)** The sentiments expressed on all gluing protests vs. environment-related protests since 2019. **(b)** Sentiments in news on environment-related gluing protests in the most prominent outlets since 2019.

We also analyzed the sentiments expressed in the reports by differentiating between (a) environment or climate-related gluing protests and (b) protests with different goals (see Figures 2a, b). The zero line represents a neutral sentiment, a positive value a positive sentiment, and a negative value a negative sentiment. Overall, throughout the period under investigation, the sentiments are mostly negative, with a few peaks of positive sentiments for both the general protests and the protests with an environmental focus when considering the sentiments across all media (see Figure 2a).

Subsequently, we also considered differences in sentiments in different outlets (see Figure 2b). Note that smoothing was performed by applying a rolling window of size 30 followed by resampling with a period of 91 days. The rolling average ensures that single data points do not determine the result of the resampled time series in periods of extreme sparsity. Sequential application of the rolling window and resampling means that not all data points are weighted equally, with data points toward the center of the resampling period being weighted more strongly. Given that most reports are about protests in the UK, the most common media reporting on the protests are also from the UK, i.e., BBC, MailOnline, The Times, The Sun, and Press Association Mediapoint. Based on previous research (compare, for example, Painter and Gavin, 2016; Cushion, 2022), these outlets can be described as follows. The BBC is a public service broadcaster that reports more news than private outlets. MailOnline is part of the Daily Mail and has a right-leaning or conservative stance. Press Association Mediapoint, or the Press Association (PA), is a private news agency with the Daily Mail and General Trust being a shareholder. The Times is considered center-right or conservative, respectively. The Sun is a tabloid newspaper and has a history of supporting the Conservative Party. Figure 2b indicates that, overall, BBC reports comparably favorably about gluing protests, with several positive sentiment periods. Two brief positive peaks can be reported as well for The Times—at the beginning and at the end of the investigated period. All other media outlets are always in the negative area, with The Sun becoming somewhat less negative during the COVID-19 protest period. MailOnline and Press Association Mediapoint are always quite negative on average.

Overall, these findings support previous findings that conservative media will report less favorably about the gluing protests. The generally negative sentiment across all media, however, is also influenced by the underlying SiEBERT model, which assigns sentences containing the word “protest” a negative value. As SiEBERT is a language model considering sequences rather than word frequencies, the exclusion of words would alter the meaning, and thus the resulting scores. Hence, we opted not to exclude them but note this expected negative sentiment as a limitation. Sentences and texts about protests are thus always rated somewhat lower by default. The differences shown between media remain valid regardless, as all values are shifted downwards.

### Reporting and the framing of gluing protests

**Figure 3:**
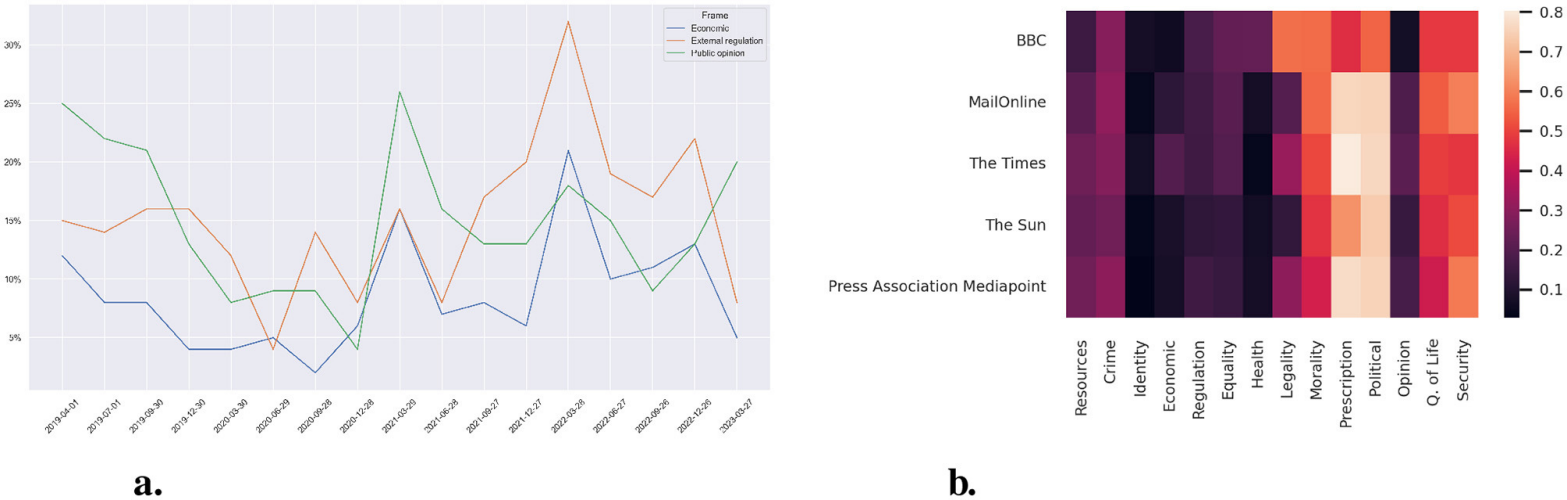
**(a)** Prevalence of the categories over time. Resampled to a period of 91 days on climate articles. Lines represent the percentage of articles including the categories. **(b)** Prevalence of the different categories in various outlets. Sources plotted against categories. Values in the percentage of articles from a source using the given category.

As a next step, we examine the occurrence of frames in our corpus using categories from a supervised BERTopic model as frame indicators. Table 1 presents the categories, their associated keywords, and initial prevalence in media reports (i.e., the proportion of reports featuring each category during the first observation period). Figure 3a focuses on protest-related categories and the economic resources frame to assess whether these key movement frames were reflected in media coverage.

**Table 1:** Keywords corresponding to the categories predicted in our corpus.

**Category**	**Prop**.	**Keywords**
Political	0.70	Climate, police, protesters, London, government, activists, protest, protests, rebellion, extinction
Policy prescription and evaluation	0.69	Climate, police, protesters, London, activists, protest, group, oil, rebellion, protests
Quality of life	0.61	Climate, change, activists, climate change, group, rebellion, extinction, London, protesters, protest
Morality	0.58	Climate, police, protesters, London, rebellion, protest, extinction, activists, extinction rebellion, protests
Security and defense	0.50	Police, protesters, climate, activists, protests, London, group, arrested, protest, government
Legality, constitutionality and jurisprudence	0.33	Police, court, protest, protesters, climate, government, London, think, protests, change
Crime and punishment	0.22	Police, court, protesters, protest, arrested, protests, London, last, activists, public
Capacity and resources	0.28	Climate, change, oil, activists, climate change, gas, energy, group, government, stop
Public opinion	0.28	Rebellion, extinction, extinction rebellion, climate, London, protesters, change, police, group, activists
Health and safety	0.18	Climate, health, change, climate change, news, police, arrested, last, world, know
Fairness and equality	0.17	Police, protesters, climate, protest, extinction, rebellion, like, think, extinction rebellion, activists
External regulation and reputation	0.17	Oil, government, gas, president, uk, energy, climate, minister, russian, ukraine
Economic	0.13	Energy, climate, oil, year, UK, government, group, last, change, gas
Cultural identity	0.10	Painting, art, climate, museum, activists, rebellion, extinction, gallery, extinction rebellion, group

Proportions are based on the occurrence in the first observation period as shown in Figure 3a.

Table 1 shows that the two most prominent categories, “Political” and “Policy prescription and evaluation,” are mentioned initially in about 70% of the reports and refer to news with keywords such as protests, police, and government. They are followed by “Quality of life” and “Morality.” Unlike the first two categories, “Quality of life” includes the keyword “change,” and “Morality” includes the movement XR, whereas neither of these two categories includes the keyword “government.” They are followed by three categories emphasizing legality and security, i.e., “Security and defense,” “Legality, constitutionality, and jurisprudence,” and “Crime and punishment.” The subsequent category, “Capacity and resources,” is most related to the frames of the protests and came up initially in about 28% of the articles in the first period of our observation. This category captures various fossil fuels and protests. Other frames that also consider fossil fuels, such as “Economic” and “External regulation and reputation,” are less common and do not focus as much on protests, but include keywords such as gas, Russia, and Ukraine. Directly related to protests are the two categories of “Public opinion” and “Cultural identity,” whereas the former reports about the protesters and the latter about specific protests. Finally, Table 1 also includes categories around topics such as health and fairness, which rank among the less common ones.

Figure 3a illustrates trends over time for the “Economic,” “External regulation,” and “Public opinion” frames. We selected these frames because their changes coincide with specific events, whereas other categories remained relatively stable or displayed more erratic patterns. The “Public opinion” frame, linked to protests, maintained a moderate overall level but declined in 2019 during the initial peak of the COVID-19 pandemic. Categories mentioning gas, Russia, or Ukraine peaked around early 2022, coinciding with the outbreak of the war.

Next, Figure 3b shows the prevalence of the categories in the most prominent media outlets for the pooled data. The colors indicate the percentage of articles that include each category. Overall, the prevalence corresponds to the findings reported in Table 1. In addition, comparing across different media shows striking similarities, apart from a few exceptions. BBC, for example, reports more often on legality and less often on the political circumstances of the protests, and The Times more often on policy prescriptions and evaluation. These small differences notwithstanding, the overall picture is a picture of similarity across media. In contrast to the sentiments, we can conclude that the reported categories are quite homogeneous across media.

## Discussion

Our research report pursued two main goals: analyzing the use of gluing as a protest tactic within climate-related movements and exploring the applicability of different NLP methods for this task. To this end, we compiled a corpus of all available English-language news articles from LexisNexis that reference gluing protests, with the earliest entry dating back to 1986. This dataset enabled us to trace the emergence and evolution of the tactic over time and across different geographic contexts, as well as examine how it has been framed in media discourse.

Regarding the development and spread of the tactic, our analysis identifies the first report of a gluing protest in the late 1980s. Initially, such actions addressed a range of issues beyond the environment. A clear shift toward environmental causes emerges from 2019 onward, with a temporary decline during the COVID-19 pandemic. These findings align with our expectations and with de Moor et al. (2021), who argues that climate protests are not a recent phenomenon. Notably, the peak of environmentally focused gluing protests occurred in 2019. While the recent wave is most prominent in the UK, the earliest reports from the 1980s originate from Australia, suggesting a different geographical origin for the tactic before its recent resurgence in Europe.

The analyses of sentiments in the media showed an overall rather negative stance, with some differences between media - which we analyzed only for the UK. BBC reported more positive (or rather less negative) than other outlets on these protests. The content of the reports is quite similar across media, with a strong focus on policy issues, whereas the prognostic frame of “what to do” is mentioned rarely. As for the framing of the protests in media, the expectation, based on Painter and Gavin (2016) and Cushion (2022), that conservative media report less favorably about these protests is confirmed. The prevalence of different frames also chimes with Entman (1993) and other views that media do select certain frames. Yet, our results showed that the selected frames (or categories) did not vary a lot across media. It was rather the tone (or sentiment) that differed considerably.

As discussed in the background section, our study is, to the best of our knowledge, the first to employ NLP methods in analyzing the historical development of gluing protests. Our findings thus add to the recent scholarship on these protests, such as Collins and Chevrette (2024); Garland (2023); Kinyon et al. (2023); and Davatkhah (2024), a distinct historical contribution by demonstrating that this form of protest dates back to at least 1986. Our study can also serve as an analytical template, illustrating how social movement research can benefit from integrating diverse NLP techniques to gain new empirical insights. Nevertheless, as we will describe below, certain methodological limitations must be acknowledged, some of which may be addressed through the use of advanced large language models (LLMs).

NLP- and LLM-based analyses are influenced by the quality and scope of the underlying data. Our study draws on a comprehensive global media database, which, while extensive, has a stronger representation of English-language sources. The results reflect a bias toward English-language coverage, as we pointed out in our results section. Expanding future research to include additional languages, also in the search terms, could offer a more encompassing perspective. At the same time, such an approach introduces new complexities, such as identifying suitable keywords and applying language-specific NLP models.

The reported negativity in the stance can be a depiction of the media's view on the protest, but can also be influenced by the models' underlying sensitivity to certain words. “Protest” is valued negatively in the SiEBERT model; hence, an analysis will return negative values in this regard. Researchers need to be aware of this limitation. Furthermore, sentiment analyses are sensitive to the number of reports considered. We ran into the challenge of several unreasonable spikes in our analyses of the media stance. These spikes occurred in periods when very few or even only a single report was printed. We thus used rolling averages for a few months to smooth the development. Here, the researcher needs to be open and clear about the underlying choices. Both limitations could be addressed by using a large-scale dataset of news reports as a baseline to compare against, e.g., reweighting and smoothing the negativity of protest reports relative to similar news without the word “protest” in it.

Besides our used NLP models, LLMs could support the inductive analysis of the data due to their capability to generate coherent summaries. They would enhance protest analysis by enabling inductive exploration, such as uncovering emergent frames and summarizing large volumes of text. Their use could be especially beneficial when expanding to multilingual corpora or less structured data sources. However, their non-deterministic nature would compromise the reproducibility of the results. Additionally, LLMs are general-purpose models not explicitly optimized for our tasks (i.e., sentiment analysis and keyword extraction). In contrast, the SiEBERT and BERTopic models employed in our study were developed especially for such tasks. Nevertheless, when used transparently and in combination with traditional NLP methods, LLMs could serve as powerful tools to deepen insights into media framing and protest dynamics.

In sum, our paper offers a first look into the development and framing of gluing protests despite the known caveats. While some of our findings align with expectations and prior research on climate protests, the analysis sheds new light on this phenomenon, such as the shift to environmental topics in 2019 and the decline during the COVID-19 period. It also underscores the applicability of NLP methods to an analysis of a current event, such as the gluing protests, using a media corpus, and provides some advice regarding how to tackle a few challenges in this regard. Future research on the development of these protests could enlarge the data base, test alternative NLP methods, and also include LLM approaches.

## Data Availability

Publicly available datasets were analyzed in this study. This data can be found here: data is derived from Lexis Nexis. The search terms are stated in the submission.
